# Medical Examiner and Western Medical Schools

**Published:** 1844-10

**Authors:** 


### MEDICAL EXAMINER AND WESTERN MEDICAL SCHOOLS.

We take great pleasure in copying from the Medical Examiner the following explanatory remarks of the Editor, called forth by the notice, in our August number, of an article in that paper, reflecting upon Western Schools of Medicine. It is our most anxious wish, not only to be on good terms with our professional brethren, whether of the press or of rival medical schools, but to see harmony prevail among the members of the profession in all the sections of our great country. We thought we saw in the remarks of the Philadelphia Editor and Professor something that was calculated to engender a bitter feeling in a large body of his brethren, and therefore alluded to them in a way which has elicited the article to which we now give place. Before finally dismissing this subject, we will take occasion to say, that we differ entirely with our New Orleans contemporary as to the tendency of the medical profession in the South. We do not believe that it has been "losing caste and respectability." True, "unworthy and incompetent members are constantly gaining admission into its ranks, and the charlatan and the empiric" are reaping every year a rich harvest. But what then? Is the same not true of our most populous, most scientific cities? We appeal to our friends in Philadelphia, in New York, in Boston, whether there is any lack of charlatans and empirics there? The race is like the goose and the vulture tribe of birds—universal. They are the true cosmopolites, but abound especially where civilization and refinement are at their height. Where science fairly culminates, there the quack finds his most genial atmosphere. Like certain diseases which attack only the luxurious and the indolent, he revels in a society where wealth and dissipation most abound—where the victims of *ennui* and nervous complaints

> "—— with sad prayers the weary doctor tease
> To name the nameless ever-new disease."

Let our New Orleans friends take courage, then, and be assured that the in-coming of the quack is but an evidence of our progress in science and refinement. It is not every constitution that can develope gout. It is not every community that can support a swarm of charlatans. We are following hard after Boston, and Paris, and London, which, long ago, was styled the "*paradise of quacks.*" Science is on the march with us. Every year the observant may see that it has gone forward. The profession is increasing in intelligence and skill. We are not retrograding—we are not stationary—but are advancing, and the host of empirics that so annoy our worthy New Orleans brethren, are but the camp-followers who hang upon the skirts of every advancing army.

But to return to the article in the Examiner, with which we shall dismiss the controversy.

"We say, then, with perfect frankness, that since the Editor thinks our remarks of the 20th of June are calculated to produce the impression, that we meant to charge either dishonor or incompetency on the *Western Medical Schools*, our remarks *were* 'unfortunate.' We had no such design. The Editors of the New Orleans Medical Journal asserted, in substance, the doctrine which we have understood to be preservingly promulgated by *one* individual in the West for years past, that those who study their profession 'in the capitals of Europe and the United States,' are unprepared to practise medicine in the Valley of the Mississippi, until they have established for themselves '*a new code of principles and practice*.' This doctrine we regarded, and still do regard, as degrading to the science. The New Orleans Journal furthermore asserts that 'the medical profession has been for some time gradually losing caste and respectability in the South—that unworthy and incompetent members are constantly gaining admission into its ranks, and the charlatan and the empiric annually find it less difficult to maintain a successful competition with the licensed practitioner.' We mentioned the fact that since the establishment of medical schools in the Valley of the Mississippi, comparatively few young men from that region seek instruction elsewhere, under the belief that there only can they learn the peculiar diseases of the South; and consequently, we inferred that if the profession in that quarter was 'gradually losing caste,' the medical 'schools in the capitals of Europe and the United States,' were certainly guiltless of contributing to the unfortunate result. Our object was to protest against what we regarded as false teaching, calculated to contract the views of ingenuous youth; but we had no intention whatever of casting any imputations of a dishonorable character upon the western or any other *schools*." Y.

